# Cytotoxic and Antiproliferative Effects of Preussin, a Hydroxypyrrolidine Derivative from the Marine Sponge-Associated Fungus *Aspergillus candidus* KUFA 0062, in a Panel of Breast Cancer Cell Lines and Using 2D and 3D Cultures

**DOI:** 10.3390/md17080448

**Published:** 2019-07-30

**Authors:** Fernanda Malhão, Alice A. Ramos, Suradet Buttachon, Tida Dethoup, Anake Kijjoa, Eduardo Rocha

**Affiliations:** 1 Institute of Biomedical Sciences Abel Salazar (ICBAS), Universidade do Porto (U.Porto), Rua de Jorge Viterbo Ferreira n 228, 4050-313 Porto, Portugal; 2 Interdisciplinary Center for Marine and Environmental Research (CIIMAR), Universidade do Porto (U.Porto), Avenida General Norton de Matos s/n, 4450-208 Matosinhos, Portugal; 3 Department of Plant Pathology, Faculty of Agriculture, Kasetsart University, Bangkok 10240, Thailand

**Keywords:** 3D cell culture, antiproliferative activity, breast cancer, cytotoxic activity, preussin

## Abstract

Preussin, a hydroxyl pyrrolidine derivative isolated from the marine sponge-associated fungus *Aspergillus candidus* KUFA 0062, displayed anticancer effects in some cancer cell lines, including MCF7. Preussin was investigated for its cytotoxic and antiproliferative effects in breast cancer cell lines (MCF7, SKBR3, and MDA-MB-231), representatives of major breast cancers subtypes, and in a non-tumor cell line (MCF12A). Preussin was first tested in 2D (monolayer), and then in 3D (multicellular aggregates), cultures, using a multi-endpoint approach for cytotoxicity (3-(4,5-dimethylthiazol-2-yl)-2,5-diphenyltetrazolium bromide (MTT), resazurin and lactate dehydrogenase (LDH)) and proliferative (5-bromo-2′-deoxyuridine (BrdU)) assays, as well as the analysis of cell morphology by optical/electron microscopy and immunocytochemistry for caspase-3 and ki67. Preussin affected cell viability and proliferation in 2D and 3D cultures in all cell lines tested. The results in the 3D culture showed the same tendency as in the 2D culture, however, cells in the 3D culture were less responsive. The effects were observed at different concentrations of preussin, depending on the cell line and assay method. Morphological study of preussin-exposed cells revealed cell death, which was confirmed by caspase-3 immunostaining. In view of the data, we recommend a multi-endpoint approach, including histological evaluation, in future assays with the tested 3D models. Our data showed cytotoxic and antiproliferative activities of preussin in breast cancer cell lines in 2D and 3D cultures, warranting further studies for its anticancer potential.

## 1. Introduction

Since cancer incidence keeps rising each year [1,2], the scientific community and pharmaceutical industry have focused their attention on the discovery of new drugs or drug adjuvants to improve the fight against this disease [3]. Consequently, one hotspot of interest for drug discovery is anticancer drugs, whose rising costs have been applied to drug research and development [3,4]. 

On the other hand, oceans not only cover 70% of the Earth’s surface, but also represent a variety of environmental niches, due to different salinities, pressures, light and oxygen levels, nutrient availability, and temperatures, which result in a great diversity of marine fauna and flora. Moreover, oceans are still an under-investigated source of bioactive compounds with medicinal benefits for human health and/or disease treatment. For this endeavor, marine-derived compounds have gained much attention in the past decades [5,6,7,8]. An example of this is the discovery of a large number of novel marine bioactive compounds with anticancer properties, leading to an increasing number of screening studies covering compounds derived from macro- and microorganisms, such as bacteria, fungi, microalgae, and seaweeds [9,10,11,12,13]. To the best of our knowledge, there are seven marine-derived drugs in clinical use for cancer treatment, e.g., cytarabine (Cytosar-U^®^) and trabectedin (Yodelis^®^), and more than twenty-three others under clinical trials, between phase II and phase III [14].

Among naturally occurring marine-derived compounds with anticancer activity, those from marine-derived fungi have been in the spotlight. Marine-derived fungi possess unique features not encountered in their terrestrial counterparts [15], and have been considered as a rich source of secondary metabolites with promising anticancer effects [12,13,16,17], representing unprecedented scaffolds for further drug design for specific modes of action [12]. Marine-derived fungi commonly exist in association with other organisms, mostly sessile invertebrates [16,18], acting as endophytes [19]. This type of association, together with the need to adapt to adverse conditions in the marine environment, contributes to a great diversity of secondary metabolites produced by marine-derived fungi [20].

**Figure 1:**
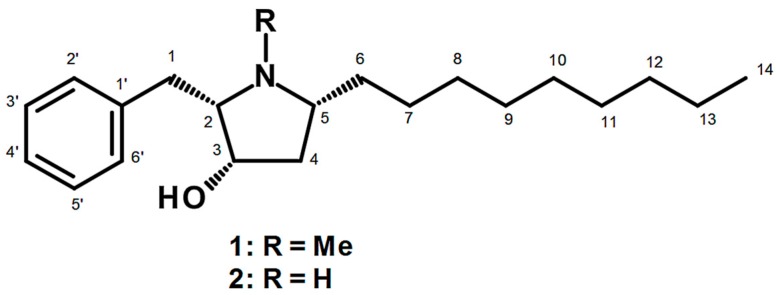
Chemical structure of preussin (**1**) and preussin C (**2**).

Recently, Buttachon et al. [21] have described the isolation and structure elucidation of—in addition to several *bis*-indolyl benzenoids—two hydroxypyrrolidine derivatives, preussin (**1**) and preussin C (**2**) (Figure 1), from the ethyl acetate extract of the culture of the marine sponge-associated fungus *Aspergillus candidus* KUFA 0062. Furthermore, all the isolated compounds were screened for their cytotoxic effect, using 3-(4,5-dimethylthiazol-2-yl)-2,5-diphenyltetrazolium bromide (MTT) assay, against eight human cancer cell lines derived from different types of tissues. Interestingly, only preussin (**1**) exhibited a significant decrease in cell viability in all the cancer cell lines tested. Consequently, we decided to explore the more in-depth effects of preussin (**1**) in breast cancer (BC) cell lines.

Breast cancer (BC) is the most commonly diagnosed cancer among women in Western countries [22,23], and a leading cause of cancer death among females [24]. Treatment of BC involves surgery, radiotherapy, and the use of anticancer drugs. However, one major problem of cancer treatments, which also applies to BC, is the multidrug resistance coupled with the toxicity of some chemotherapeutics [25,26]. The emergence of drug resistance triggers the search for new drugs or drug adjuvants and, simultaneously, the need for a better understanding of the molecular mechanisms involved in drug resistance [27,28]. Accordingly, a search for compounds that are aimed at different therapeutic targets and/or that potentiate the existing established drugs with minimal, or at least decreased, toxicity towards normal cells has become a priority [29].

Cell lines have greatly contributed to a better understanding of BC molecular mechanisms. Nonetheless, some authors have stressed the importance of choosing an appropriate cell line panel as an experimental model with specific sub-characteristics that could influence the responses to different compounds of potential therapeutic interest [30,31]. Breast cancer is very heterogeneous in terms of histological types and clinical outcomes, namely having different patterns of positivity for estrogen and progesterone receptors, as well as for the expression of the oncogene human epidermal growth factor receptor 2 (HER-2). These different characteristics are fundamental in determining therapeutic approach [32].

Accordingly, for this study, we selected four human breast cancer cell lines with some characteristics corresponding to BC subtypes: (i) MCF7, which has positive estrogen and progesterone receptors and is negative for HER-2 overexpression (ER+, PR+, HER-2–), corresponding to the most common BC type—Luminal A [30,33]; (ii) SKBR3, a negative for estrogen and progesterone receptors and positive for HER-2 overexpression (ER–, PR–, HER-2+), representing the HER-2 subtype [30,33]; (iii) MDA-MB-231, a ‘triple negative cell line’ (ER–, PR, HER2–), corresponding to the basal-type breast carcinoma cell [30,33]; and (iv) MCF12A, which is a non-tumor breast cell line [30,34].

Nowadays, it is well established that cell culture research performed in monolayer (2D) has a low predictive capacity, especially in the field of drug discovery where great investments have been made each year [35]. The lack of three-dimensional (3D) geometry is associated with less intercellular interactions, and different microenvironments which result in different biochemistry, gene expression, and drug metabolism [36,37]. All these differences partially explain why many drugs tested in 2D cultures fail when tested in in vivo models or in clinical trials [38,39]. Three-dimensional (3D) breast cell cultures recapitulate some of the physiological and architectural aspects of breast epithelium [40], which may represent a model closer to the in vivo than the 2D cultures.

Based on the promising data of our recent research [21], the aim of this study was to specifically assess the in vitro anticancer activity of preussin (**1**), namely cytotoxic and antiproliferative effects, in a panel of three breast cancer cell lines and one non-tumor breast cell line, cultured in 2D and 3D culture models.

## 2. Results

### 2.1. Cells Exposure in 2D

#### 2.1.1. Analysis of Cell Viability—MTT Assay

**Figure 2:**
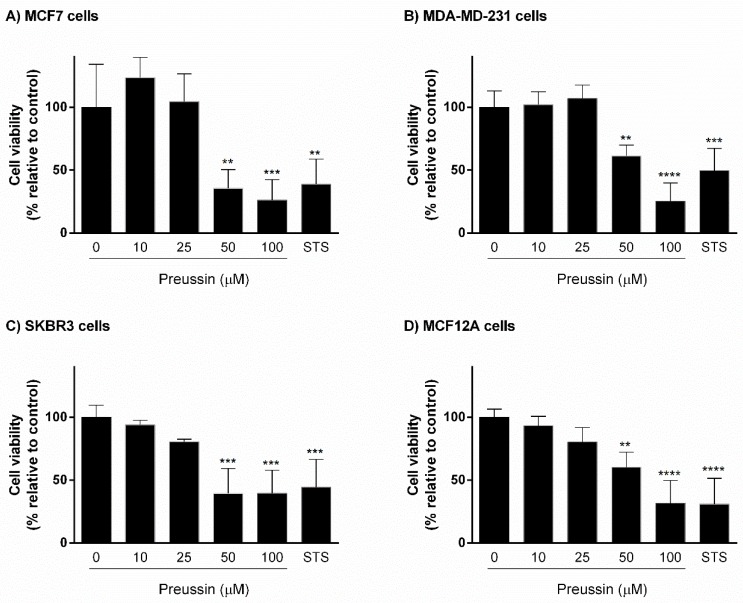
Effect of preussin (**1**), at 10, 25, 50, and 100 µM, on cell viability in 2D culture. (**A**) MCF7, (**B**) MDA-MB-231, (**C**) SKBR3, and (**D**) MCF12A cells after 72 h of incubation, assessed by 3-(4,5-dimethylthiazol-2-yl)-2,5-diphenyltetrazolium bromide (MTT) assay. Cells treated with 0.1% DMSO (solvent; SC) and staurosporine (STS; 1 µM) were included as negative and positive controls, respectively. The results were expressed as the percentage of cell viability, relative to negative control, and are presented as mean ± standard deviation (SD) of four independent experiments (two duplicates per replica). (*** *p* < 0.01; *** *p* < 0.001; **** *p* < 0.0001).

Cells were exposed for 72 h either to preussin (**1**) at different concentrations (10, 25, 50, and 100 μM), or to staurosporine (STS) (1 μM), a positive control, for apoptosis induction [41,42]. Culture medium containing only solvent (SC) (medium with 0.1% DMSO, v/v) was used as a negative control. Cells exposed to preussin (**1**) at 50 and 100 µM showed significant decrease in cell viability in the three cancer cell lines (MCF7, MDA-MB-231, and SKBR3) and in the non-tumor cell line (MCF12A). STS decreased cell viability to less than 50%, in relation to the control, in all cell lines tested (Figure 2).

#### 2.1.2. Analysis of Cell Proliferation—5-bromo-2′-deoxyuridine (BrdU) Assay

**Figure 3:**
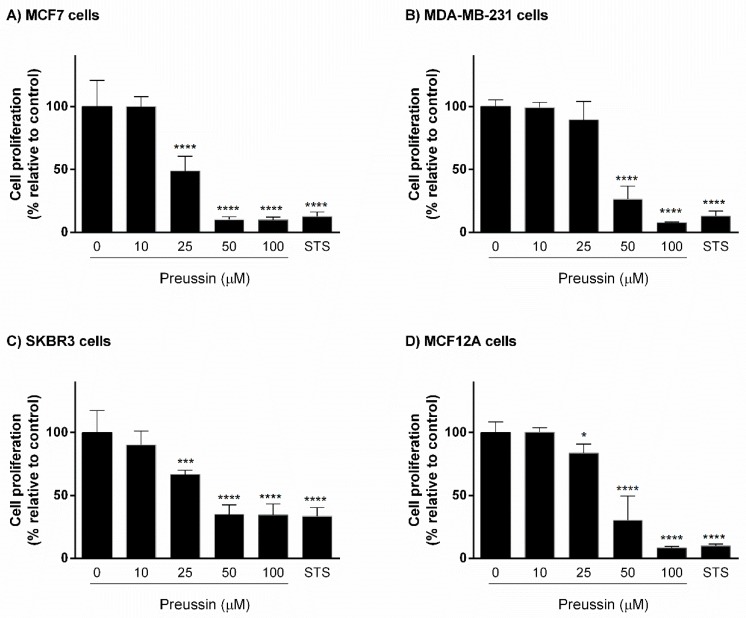
Effect of preussin (**1**), at 10, 25, 50, and 100 µM, on cell proliferation in 2D culture. (**A**) MCF7, (**B**) MDA-MB-231, (**C**) SKBR3, and (**D**) MCF12A cells after 72 h of incubation, assessed by 5-bromo-2′-deoxyuridine (BrdU) assay. Cells treated with 0.1% DMSO (SC) and STS (1 µM) were included as negative and positive controls, respectively. The results were expressed as the percentage of cell proliferation, relative to negative control, and are presented as mean ± SD of four independent experiments (two duplicates per experiment). (* *p* < 0.05; *** *p* < 0.001; **** *p* < 0.0001).

Cells were exposed for 72 h either to preussin (**1**) at different concentrations (10, 25, 50, and 100 μM) or to STS (1 μM). Preussin (**1**) induced a decrease of cell proliferation in all cell lines. In MCF7 and SKBR3 cells, as well as in MCF12A, preussin (**1**) at 25 µM was able to significantly reduce cell proliferation. In contrast, in MDA-MB-231, preussin (**1**) only at 50 µM significantly inhibited cell proliferation. At 50 µM, preussin (**1**) led to a decrease of cell proliferation below 50%, in relation to the control, in all cell lines. STS inhibited cell proliferation in all cell lines, with less potency toward SKBR3 (Figure 3).

#### 2.1.3. Cell Morphology

When observed in the phase contrast microscopy, SC groups showed nearly 90% of confluence. Cells exposed to STS and preussin (**1**) at 50 and 100 µM revealed morphological alterations, with vacuolization of the cytoplasm, loss of cell adhesion leading to cell detachment, and, consequently, lower density (data not shown). 

### 2.2. Cells Exposure in 3D

#### 2.2.1. Analysis of Cell Viability

From the results obtained in 2D culture, we selected the concentrations of preussin (**1**) that exhibited more pronounced effect on cell viability and proliferation (50 and 100 µM). The assessment of the cytotoxic effect of preussin (**1**) in 3D culture was performed using three cell viability assays: MTT, resazurin, and lactate dehydrogenase (LDH).

##### MTT Assay

**Figure 4:**
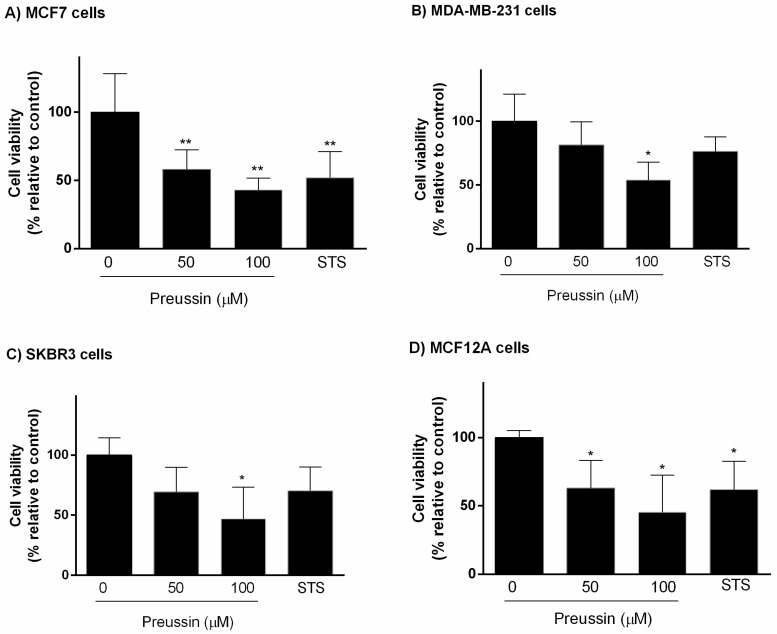
Effect of preussin (**1**), at 50 and 100 µM, on cell viability in 3D culture. (**A**) MCF7, (**B**) MDA-MB-231, (**C**) SKBR3, and (**D**) MCF12A cells after 96 h of incubation, assessed by MTT assay. Cells treated with 0.1% DMSO (SC) and STS (1 µM) were included as negative and positive controls, respectively. The results were expressed as the percentage of cell viability, relative to negative control, and are presented as mean ± SD of four independent experiments (two duplicates per experiment). (* *p* < 0.05; ** *p* < 0.01).

Cells were exposed for 96 h to preussin (**1**) at two concentrations (50 and 100 μM) or STS (1 μM). Preussin (**1**) at 50 μM revealed significant effect on cell viability only in MCF7 and MCF12A cell lines, decreasing cell viability, while at 100 μM, it decreased cell viability in all cell lines. Similar to preussin (**1**) at 50 μM, STS caused a significant decrease in cell viability only in MCF7 and MCF12A cell lines, with no statistically significant effect on cell viability in MDA-MB-231 or SKBR3 cell lines (Figure 4).

##### Resazurin Assay

**Figure 5:**
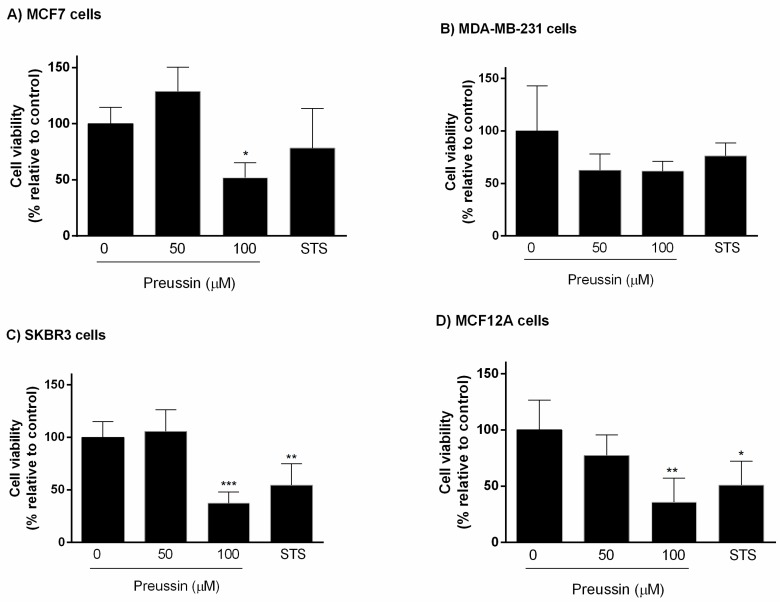
Effect of preussin (**1**), at 50 and 100 µM, on cell viability in 3D culture. (**A**) MCF7, (**B**) MDA-MB-231, (**C**) SKBR3, and (**D**) MCF12A cells after 96 h of incubation, assessed by resazurin assay. Cells treated with 0.1% DMSO (SC) and STS (1 µM) were included as negative and positive control, respectively. The results were expressed as the percentage of cell viability, relative to negative control, and are presented as mean ± SD of four independent experiments (two duplicates per experiment). * *p* < 0.05; ** *p* < 0.01; *** *p* < 0.001).

Cell viability was also investigated using the resazurin reduction assay. Figure 5 shows the effect of preussin (**1**) on cell viability in 3D culture. After 96 h of exposure to preussin (**1**), either at 50 or 100 µM, significant decreases in cell viability were detected at 100 µM in MCF7, SKBR3, and MCF12A cell lines. As for MDA-MB-231 cells, no differences were observed under preussin (**1**) influence. STS did not cause any significant impact on MCF7 or MDA-MB-231 cells, but decreased viability of SKBR3 and MCF12A cells.

##### LDH

**Figure 6:**
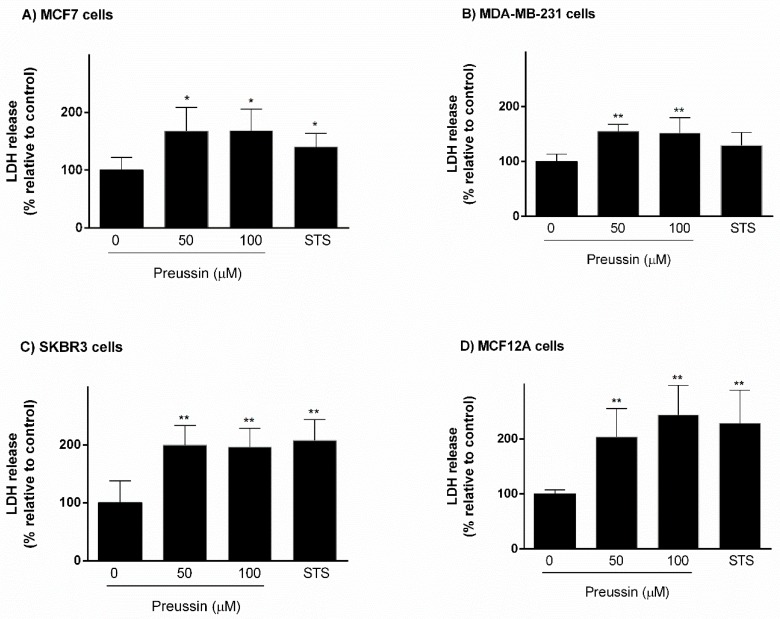
Effect of preussin (**1**), at 50 and 100 µM, on cell viability in 3D culture. (**A**) MCF7, (**B**) MDA-MB-231, (**C**) SKBR3, and (**D**) MCF12A cells after 96 h of incubation, assessed by lactate dehydrogenase (LDH) assay. Cells treated with 0.1% DMSO (SC) and STS (1 µM) were included as negative and positive controls, respectively. The results were expressed as the percentage of LDH release, relative to negative controls, and are presented as mean ± SD of four independent experiments (two duplicates per experiment). (* *p* < 0.05; ** *p* < 0.01).

The third endpoint used for evaluating cytotoxic effect of preussin (**1**) in 3D culture was the LDH assay. Preussin (**1**), at 50 and 100 µM, induced an increase in LDH release in relation to negative controls, corresponding to a decrease in cell viability in all cell lines. In the case of SKBR3 and MCF12A cells, preussin (**1**) led to nearly 100% increase in LDH release in comparison to controls. The effects of STS and preussin (**1**) on LDH release were very similar in MCF7, SKBR3, and MCF12A cell lines. However, in MDA-MB-231, STS did not demonstrate a significant effect on LDH release (Figure 6).

#### 2.2.2. BrdU Proliferation Assay

**Figure 7:**
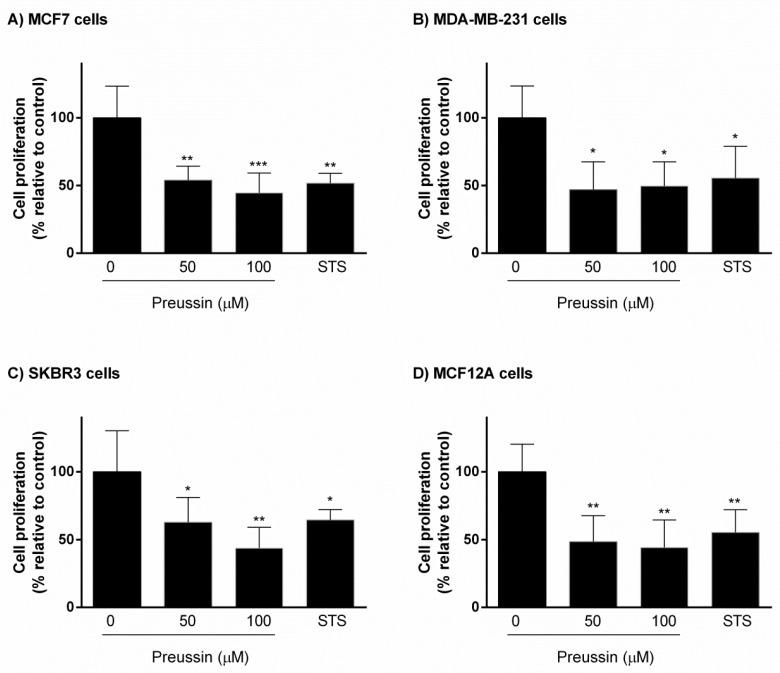
Effect of preussin (**1**), at 50 and 100 µM, on cell proliferation in 3D culture. (**A**) MCF7, (**B**) MDA-MB-231, (**C**) SKBR3, and (**D**) MCF12A cells after 96 h of incubation, assessed by BrdU assay. Cells treated with 0.1% DMSO (SC) and STS (1 µM) were included as negative and positive controls, respectively. The results were expressed as the percentage of cell proliferation, relative to negative control, and are presented as mean ± SD of four independent experiments (two duplicates per experiment). (* *p* < 0.05; ** *p* < 0.01***; *p* < 0.001).

When compared to controls, preussin (**1**) at 50 and 100 µM inhibited cell proliferation at approximately 50% in all cell lines cultured in 3D (Figure 7). STS promoted similar results in magnitudes comparable to those of preussin (**1**).

#### 2.2.3. Analysis of Multicellular Aggregates (MCAs) Morphology in 3D Culture

##### Stereomicroscopy Analysis

Plates containing MCAs were observed daily in a stereomicroscope with dark field. During the formation time, it was possible to observe cell aggregation to form the MCAs. The type of MCA was dependent on the cell line, but the MCAs of the same cell line were quite similar.

**Figure 8:**
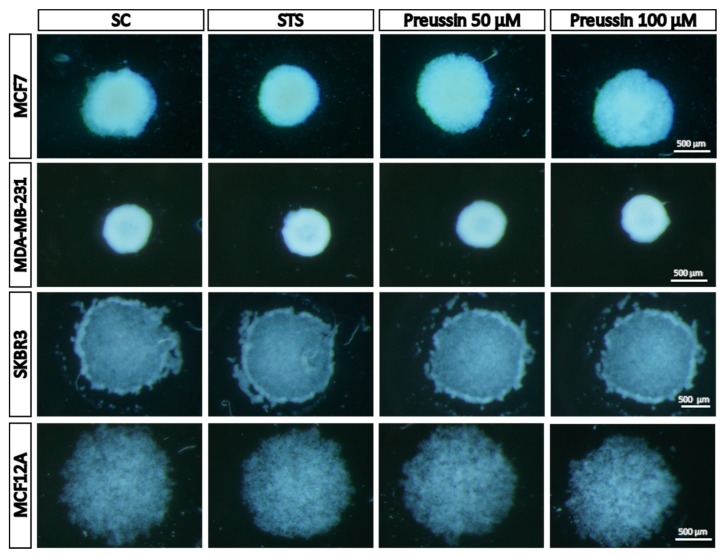
Representative morphology of multicellular aggregates (MCAs) in 3D culture, photographed in a stereomicroscope. The MCAs of MCF7, MDA-MB-231, SKBR3, and MCF12A after 96 h of incubation with preussin (**1**) (50 and 100 µM). Cells treated with 0.1% DMSO (SC) and STS (1 µM) were included as negative and positive controls, respectively.

Figure 8 shows a typical morphology of MCAs from the four cell lines in the solvent control and exposed conditions after 72 h of formation, plus 96 h of exposure. After MCA formation (before the exposure—data not shown), MCF7 and MDA-MB-231 cells formed MCAs with round shapes, the MDA-MB-231′s MCAs being more compact than those of the MCF7. As for MCF12A and SKBR3 cell lines, cells did not aggregate so obviously, even after seven days in culture, ultimately forming loose MCAs. There were no obvious differences in MCAs before and after the exposure. As can be observed in Figure 8, after 96 h of exposure, either to preussin (**1**) or to STS, the shape and size of the MCAs were similar to those of the negative control (SC).

##### Multicellular Aggregate Measurements

**Figure 9:**
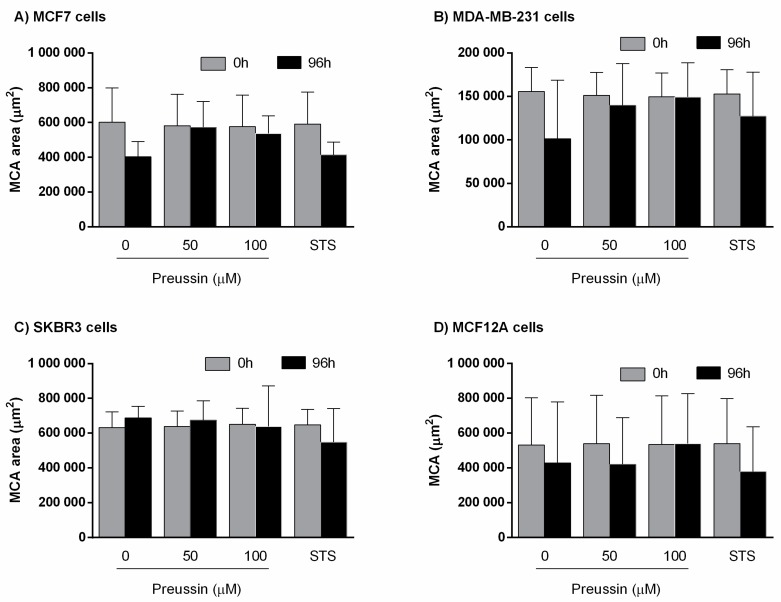
Multicellular aggregates (MCAs) areas (µm^2^) in 3D culture. (**A**) MCF7, (**B**) MDA-MB-231, (**C**) SKBR3, and (**D**) MCF12A before exposure (t1) (gray bars) and after 96 h of exposure (t2) (black bars). Cells were exposed for 96 h to preussin (**1**) (50 and 100 µM). Treated cells with 0.1% DMSO (SC) and STS (1 µM) were included as negative and positive controls, respectively. Results are presented as mean ± SD of four independent experiments (12 duplicates per replica).

For their measurements, MCAs were photographed at two different times: (1) After 72 h of spheroids formation (before the exposure); and (2) after 96 h of exposure (total of seven days in culture). Using these pictures, the software performed the segmentation, creating binary images for further data extrapolation. The areas obtained with the AnaSP software [43] are presented in Figure 9. Data from the sphericity and solidity of the MCAs are given in Figure S1.

There were no significant differences between MCAs’ areas of *t*1 = 0 h (before exposure), corresponding to 72 h of MCA formation, and *t*2 = 96 h, corresponding to after 96 h of exposure.

In the same manner, there were no differences between SC and any of the other conditions (Figure 9).

##### Morphology

After 96 h of exposure, MCAs were fixed, processed for paraffin embedding, and sectioned for hematoxylin-eosin (HE) staining and immunocytochemistry analysis (ICC).

**Figure 10:**
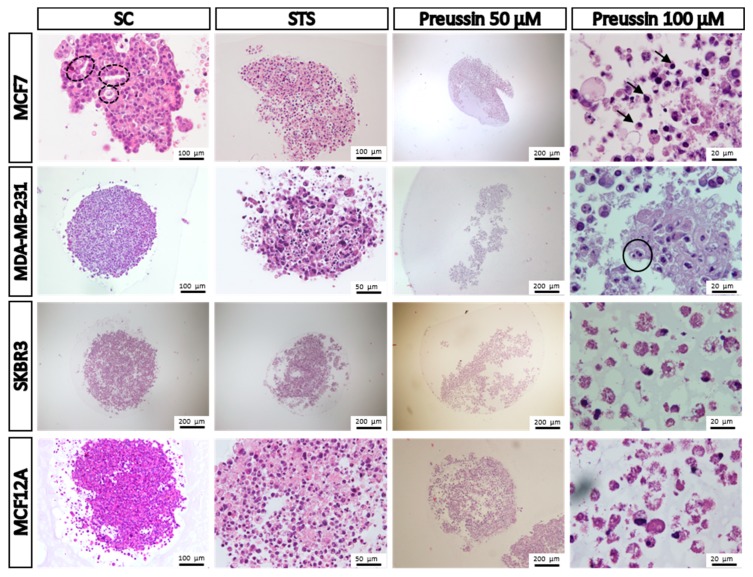
Representative morphology of the multicellular aggregates (MCAs) of MCF7, MDA-MB-231, SKBR3m and MCF12A after 96 h of incubation with preussin (**1**) at 50 and 100 µM. Cells treated with 0.1% DMSO (SC) and STS (1 µM) were included as negative and positive controls, respectively. Hematoxylin-eosin (HE) staining. Arrowhead: Dense chromatic and nuclear shrinkage; dashed line circle: Acinar-like structures; straight line circle: Nuclear fragmentation.

Through the observation of MCAs stained with HE, at the microscopic level, it was possible to note that different cell lines (SC groups) displayed different levels of cell compaction, similar to those observed by stereomicroscopy (compare Figure 8 with Figure 10). Also, MCAs from SC groups revealed a more intact structure than those exposed to preussin (**1**). This is explained by the fact that when MCAs from preussin-treated cells were transferred either to a flat-bottom plate for the viability assays or to a tube for fixation, they tended to easily disintegrate, forming a cell suspension (see preussin 50 µM exposed cells in Figure 10).

In all sectioned MCAs (eight per condition and per cell line), there was only one (from a SC group of MCF7 cells) that showed a central necrotic core. In the case of the groups exposed to STS or preussin (**1**), most MCAs cells showed damaged morphology, indicating cell death. The degree of damage was more severe after exposure to preussin (**1**) than to STS; however, there were no differences between the two tested concentrations of preussin (**1**). Cells from MCAs exposed to STS and preussin (**1**) in the four cell lines revealed some of the typical features of apoptotic cells: Cell shrinkage, nuclear condensation, chromatin margination, karyorrhexis, cell detachment, and apoptotic bodies [44,45]. From a histological point of view, the number of cells with a morphology compatible with cell death is evidently higher in the preussin (**1**)-exposed groups. In MCAs of MCF7 cells (SC group), it was quite common to find acinar-like structures with lumina that resemble those of the normal mammary gland. Indeed, in those cases, groups of cells within the MCAs were organized so that they formed irregular shaped lumina, sometimes containing apoptotic cells [46] (Figure 10).

##### MCA Immunocytochemical Analysis

**Figure 11:**
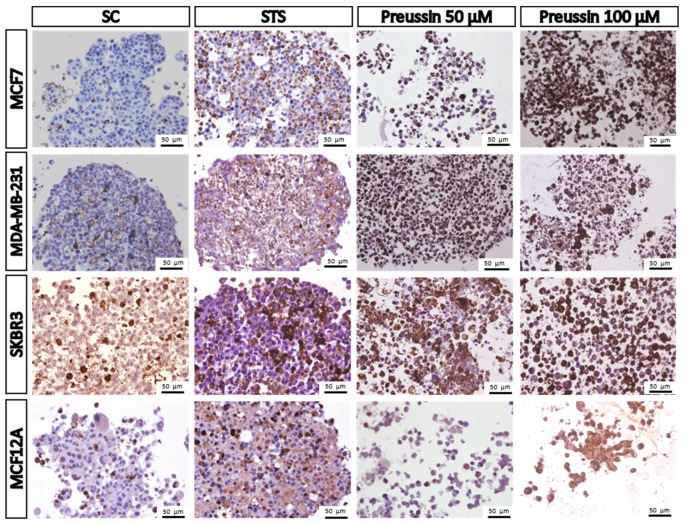
Representative images of immunostaining against caspase-3 in multicellular aggregates (MCAs) cells. MCF7, MDA-MB-231, SKBR3, and MCF12A MCAs after 96 h of incubation with preussin (**1**) at 50 and 100 µM. Cells treated with 0.1% DMSO (SC) and STS (1 µM) were included as negative and positive controls, respectively. The brown staining in the cytoplasm corresponds to the immunolocalization of caspase-3 protein, as revealed by the diaminobenzidine chromogen.

**Figure 12:**
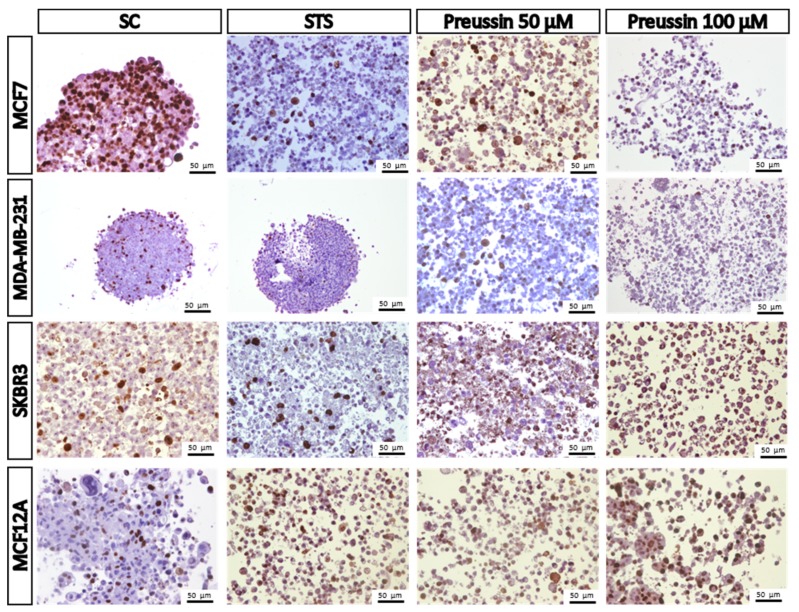
Representative images of immunostaining against ki67 of multicellular aggregates (MCAs). MCF7, MDA-MB-231, SKBR3, and MCF12A MCAs after 96 h of incubation with preussin (**1**) at 50 and 100 µM. Cells treated with 0.1% DMSO (SC) and STS (1 µM) were included as negative and positive controls, respectively. The brown staining in the nuclei corresponds to the immunolocalization of ki67 protein, as revealed by the diaminobenzidine chromogen.

In order to support the basic morphological information, the immunocytochemistry (ICC) technique was performed using two different antibodies: Anti-caspase-3 and ki67. Caspase-3 is considered a biochemical marker of cell apoptosis [47,48] while ki67 is a classical marker for cell proliferation [49,50]. The results showed that there were some positive cells for caspase-3 in all the MCAs of the SC groups. These positive cells were randomly distributed for all the MCAs, without any specific localization (Figure 11), except for the lumen of acinar-like structures found in MCF7, where it was quite common to find apoptotic cells (Figure 11). However, when the number of caspase-3-stained cells in the SC group was compared to those in the drug-exposed groups, it was found that the number of apoptotic cells in the drug-exposed group was clearly higher. The immunostaining for ki67 was also distributed all along the MCAs, without having any preferential area (Figure 12).

**Figure 13:**
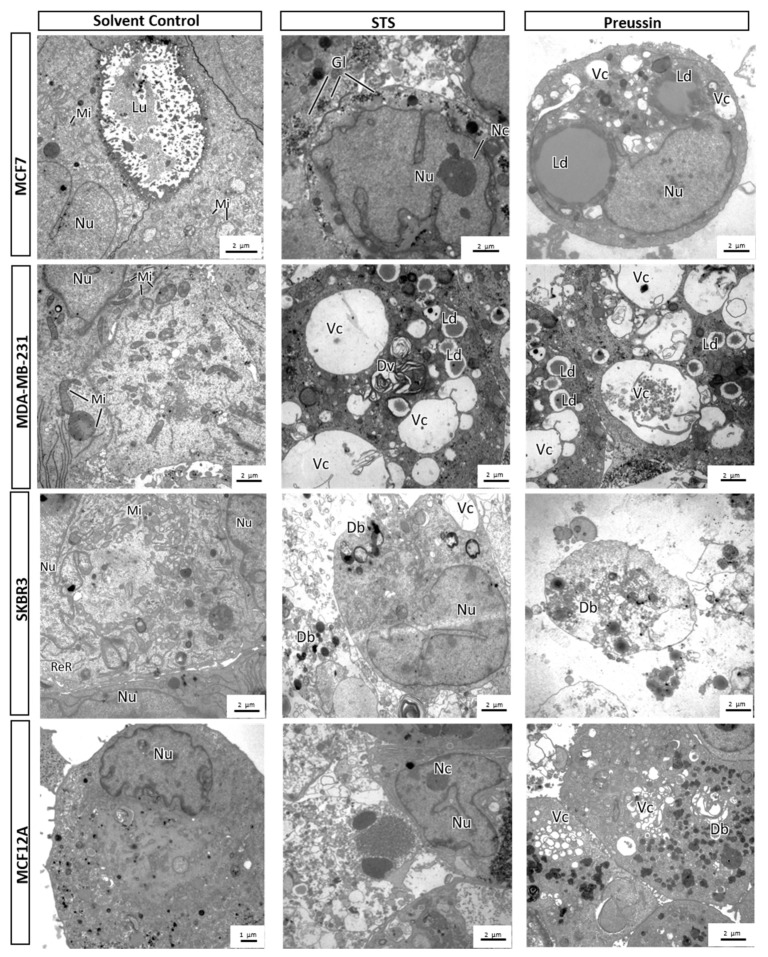
Representative pictures of electron microscopy analysis of multicellular aggregates (MCAs). MCF7, MDA-MB-231, SKBR3, and MCF12A MCAs after 96 h of incubation with preussin (**1**) (50 and 100 µM). Cells treated with 0.1% DMSO (SC) and STS (1 µM) were included as negative and positive controls, respectively. Db: Dense bodies; Dv: Degenerative vesicles; Gl: Glycogen; Ld: Lipid droplets; Lm: Lumen; Mi: Mitochondria; Nc: Nucleoli; Nu: Nucleus; ReR: Rough endoplasmic reticulum; Vc: Vacuole.

Immunostaining for caspase-3 showed a pattern of increasing immunostained cell number (SC ˂ STS ˂ preussin (**1**)) which was inversely proportional to that of ki67, where a higher number of positive cells was found in the SC group (SC > STS > preussin (**1**)) (Figure 12 and Figure 13, respectively). Moreover, morphological observations did not reveal any differences between the two tested concentrations of preussin (**1**). Both preussin-exposed conditions showed a great number of cells marked with caspase-3 (more than 80% of all cells) and some cells revealed ki67 immunostaining (Figure 11 and Figure 12, respectively). Judging by the proportion of cells positive for ki67, MCF7 seems to be the most proliferative of the four cell lines, where approximately 50% of their cells, in MCA form, were positive for ki67 (See Figure 12).

##### MCA Electron Microscopy

The ultrastructural morphology of MCAs revealed that all cell lines in the SC groups displayed some common features, such as euchromatic, irregular shape nuclei with prominent nucleoli, and an abundant presence of mitochondria, in contrast with rare rough endoplasmic reticulum (RER) and Golgi apparatus profiles. In MCF7, MCF12A, and SKBR3, many cells showed glycogen deposits (not seen in all images) and lipid droplets (Figure 13).

All cell lines treated with STS and preussin (**1**) (at both concentrations) showed an increased number of enlarged pleomorphic vacuoles (some with concentric degenerative appearance) and dense bodies in the cytoplasm. In the same manner, a great number of nuclei with peripheral coarse chromatin condensation and karyorrhexis was observed. Lipid droplets and vesicles were also increased in drug-treated cells (Figure 13).

## 3. Discussion

To the best of our knowledge, this is the first study on cytotoxicity and antiproliferative effects of preussin (**1**), a hydroxypyrrolidine derivative, isolated from the marine-derived *A. candidus* KUFA 0062, in a panel of three BC cell lines (MCF7, SKBR3, and MDA-MB-231) which correspond to three biological and immunophenotypic distinct types of BC, and one non-tumor breast cell line (MCF12A), with a comparison of its effects in 2D and 3D cultures. The 2D culture approach was used initially to obtain the results, which allowed us to proceed to the 3D model, a more sophisticated and physiologically relevant type of culture, which generates more predictive data than monolayer cultures [38,51].

The screening of the cytotoxic and antiproliferative effects of preussin (**1**) was performed in 2D culture, using MTT and BrdU assays, respectively. STS, an apoptosis inductor [42], was used as a positive control. Preussin (**1**) at 50 µM, and STS, decreased cell viability in all cell lines. In relation to the effects on cell proliferation, preussin (1) at 25 µM was enough to inhibit cell proliferation in MCF7, MCF12A, and SKBR3, but not in MDA-MB-231, where inhibition only occurred at 50 µM. Regarding STS, it had negative effects in all cell lines.

After confirming that preussin (**1**) could elicit cytotoxic and antiproliferative effects in 2D culture, our next goal was to verify if such effects were maintained when cells are in a 3D culture model. For this, we selected concentrations that showed significant effects in 2D (50 and 100 µM). Several studies have shown that cancer cell lines, including BC cell lines, are less sensitive to anticancer agents in 3D cultures [39,52]. Accordingly, it is known that there is a refractory effect towards drugs when tested in 3D cultures, where cells are less susceptible to the impact, so that the concentrations that cause similar effects in 3D cultures are higher than in 2D cultures [53]. Despite being closer to the in vivo setting, 3D models are far from routine use, and studies continue to be mainly conducted in 2D cultures.

Concerning 3D culture of cell lines, a variety of methodologies has been developed [34,54,55]. However, the simple and effective method of seeding the cells in ultra-low attachment plates with conical shape was opted in this study. This method produced uniform sized 3D aggregates within the same cell line, as can be verified by morphometric measurements, and in accordance with what was described in the literature [56]. This uniformity is quite important when screening compounds for cytotoxicity [57].

The 3D multicellular structures can differ according to the cell line, methodology used, cell density, and time in culture [58]. Also, they have been named differently: Spheroids [53,56,59]; tumor spheroids [58,60] (even per organ: Breast/mammary cancer spheroids [61,62] or mammospheres [63,64]); microtissues [65]; multicellular tumor spheroids [55,66,67]; and mixed terminology to 3D aggregates/spheroids [53]. Herein, according to the observed morphology, the term ‘multicellular aggregates (MCAs)’ is used for the 3D multicellular structures, since they are 3D aggregates without a true spheroid shape, being a bit flattened and resembling a ‘pancake’.

In this study, after 72 h of seeding, MCF7 and MDA-MB-231 formed tight MCAs, while MCF12A and SKBR3 produced looser MCAs. These observations were in agreement with those reported in previous studies [58,68]. These morphological characteristics were maintained during the exposure to preussin (**1**) and STS or in the SC. At 72 h (before exposure) (*t* = 0), and after 96 h of exposure (*t* = 96), MCAs were measured using AnaSP software, which allowed the assessment of different parameters in a very limited amount of time without resorting to further instrumentation [35,43]. Spheroid area has been considered as the most informative parameter [69], and changes in MCAs’ morphology and size (together with other endpoints) have been reported as a consequence of the drug’s effect [69,70]. As there was no difference between MCAs of the SC at the two studied times (*t* = 0 and *t* = 96 h), it was concluded that MCAs’ areas were stable along the exposure time in solvent control conditions. With regard to preussin (**1**) exposure, there were no significant differences when compared with SC. This means that, in our study, the area parameter did not reflect any drug effects. At the end of the exposure time, MCAs can be harvested and then analyzed by colorimetric, fluorescence, and luminescence assays using a plate reader [36]. Accordingly, we studied the cytotoxic and antiproliferative effect of preussin (**1**) by the same assays used in 2D culture (MTT and BrdU), together with two more assays for cytotoxicity (LDH and resazurin).

The MTT assay in 3D culture showed that MDA-MB-231 and SKBR3 were more resistant to the effects of preussin (**1**) and STS. Concerning cytotoxicity in MCAs, the LDH assay showed that preussin (**1**) at 50 µM caused an increase in LDH release into the extracellular medium (in relation to the control) in all cell lines. When comparing MTT and LDH results, the decrease in cell viability in MTT assay correlates well with an increase in LDH release. It is noteworthy to point out that a concentration of 50 µM of preussin (**1**) was enough to cause significant differences in the LDH assay; however, in the MTT assay, especially in the MDA-MB-231 and SKBR3 cells, a concentration of 100 µM of preussin (**1**) was necessary to cause the same effect. In relation to the resazurin reduction assay, differences were detected only after exposure to 100 µM of preussin (**1**) in MCF7, MCF12A, and SKBR3 cells; however, no differences were detected in MDA-MB-231 cells at this concentration.

Globally, in the comparison of all the endpoints of cell cytotoxicity in 3D culture, all assays pointed to a cytotoxic effect of preussin (**1**), varying only in the concentration for which the effect was significantly detected. The effect of STS varied according to the assay method. It has also been reported that STS can be used up to 10 µM; however, in this case a complete dissociation of the spheroid occurred at this concentration [71]. In future studies with MCAs, we suggest using a concentration between 1 and 10 µM.

Considering the characteristics of the cell lines used in this study, different results are logically explainable. MCF7 is a cell line with positive estrogen receptors, which is more responsive to therapeutics [29], while the MCF12A cell line has been described as non-tumorigenic. Both cell lines were more susceptible to the tested compounds. The use of normal cell lines from the same organ/tissue in screening studies of new drugs have been considered important, by some authors, to offer hints about the toxicity of these compounds in non-tumor cells [17]. However, this importance has also been questioned by others, who defend that a compound should not be rejected for further testing just because of its toxicity in “normal cell lines” [72]. This concept is justified by the fact that the clinical relevance of the toxicity is not towards cells of the same tissue, but, conversely, towards other type of tissues, namely fast-proliferating cells—i.e., those that cannot regenerate (like cardiomyocytes and neurons)—and cells from metabolic organs where drugs are metabolized or excreted [72]. SKBR3 cells that have HER-2 overexpression, and BC, with this characteristic, normally progress more aggressively than those with normal expression [73]. In accordance with this concept, the SKBR3 cell line viability was affected more with preussin (**1**) at the highest concentration (100 µM). The cell line which was less responsive to exposure was the triple-negative MDA-MB-231, a representative of the most aggressive BC subtype, which is harder to treat and more likely to metastasize [74].

Taking into account the results from BrdU proliferation assay, preussin (**1**) (at respective tested conditions) inhibited cell proliferation in all cell lines, cultured in 2D or in 3D, with lower concentrations in 2D.

All the above findings were corroborated by morphological analysis (optical and electron microscopy). The cytotoxic effects of preussin (**1**), revealed by histology and electron microscopy observation, were very clear and showed a higher extent of damage than that revealed by the data obtained from cytotoxicity assays. The MCAs exposed to preussin (**1**) had a normal appearance with intact structure when photographed in the plates, before the collection for optical and electron microscopy. However, when manipulated, they tended to disrupt upon pipette mechanical manipulation. This was not observed in MCAs of the SC. When observing the cells under the microscope, it was understandable why these MCAs disaggregate, as the cells’ morphology was severely altered by exposure to compounds, STS and especially preussin (**1**). In MCAs stained with HE, all typical aspects of apoptosis—i.e., cytoplasmic and nuclear condensation, nuclear fragmentation, and hyper eosinophilic cytoplasm—were observed [75]. In addition to a simple morphological study, the ICC technique against caspase-3 (apoptosis marker) [47,76] and ki67 (proliferation marker) [77] was also performed. Curiously, the more commonly described phenotype for spheroids, with a proliferative outer layer, a quiescent zone, and a necrotic core [38,78], was not found in the MCAs. MCAs of MCF7 in the SC group revealed acinar-like structures with lumina [65,79], inside which caspase-3 positive cells were detected. This is in line with the lumen formation process of acini, where apoptotic clearance of the inner cells occurs [59,80].

The ICC technique provided useful information in relation to cell death and cell proliferation. For the drug-exposed groups, especially for preussin (**1**) at both concentrations, most cells stained for caspase-3. In accordance with the cells’ morphological characteristics, this data is compatible with ongoing cell death. In this scenario, it would be normal to find just a few or no cells under cell division. In fact, besides a huge number of cells stained with caspase-3, ki67 positive cells were not that insignificant. The number of ki67-positive cells was clearly decreased in preussin (**1**)-exposed MCAs in relation to the control. However, we found more ki67-positive cells in preussin (**1**)-exposed MCAs than expected. The results obtained from the ICC against ki67 can be compared with those obtained with the BrdU proliferation assay. Ki67 is a protein expressed in almost all phases in the cell cycle: S, G1, G2, and M phases, but not in G0 [77]. On the other hand, BrdU proliferation assay is a technique in which BrdU is incorporated into the DNA in the S phase of the cell cycle [81]. In this circumstance, the number of cells immunostained with ki67 was much lower in the drug-exposed groups than in the SC groups, which were similar to the data obtained from the BrdU proliferation assay.

Our opinion is that the anticancer effects should be assessed using multiple endpoints, as we verified that the results obtained from different assays can be quite different. Morphology can also contribute to a better understanding of the degree of damage affecting the exposed cells.

Overall, the toxicity and antiproliferative data unveiled by this study, using cancer cell lines representative of biologically distinct BC, suggest that preussin (**1**) can represent a potential scaffold for the development of a future anticancer drug. The data obtained from this study clearly illustrates the importance of conducting comparative studies using 2D and 3D culture models, with the effects observed in the latter reinforcing the potential of preussin (**1**), warranting more research to explore the in vitro cytotoxicity and mechanisms of action. As a next research step, molecular biology tools should help to unveil the possible signaling pathways involved in the cytotoxic and antiproliferative effects of preussin (**1**).

## 4. Materials and Methods

### 4.1. Cell Lines Cultivation

MCF12A and MDA-MB-231 cell lines were purchased from the American Tissue Culture Collection (ATCC). MCF7 was acquired from the European Collection of Authenticated Cell Cultures (ECACC). The SKBR3 cell line was kindly provided by Professor Carmen Jerónimo of the Portuguese Oncology Institute - Porto. MCF7, MDA-MB-231, and SKBR3 were cultivated in Dulbecco’s Modified Eagle’s Medium high glucose (DMEM) without glutamine and without phenol red, supplemented with 10% Fetal Bovine Serum (FBS) and 1% penicillin/streptomycin. MCF12A was cultivated in a mixture of DMEM/F12 medium without phenol red, and supplemented with 20 ng/mL human epidermal growth factor, 100 ng/mL cholera toxin, 0.01 mg/mL insulin, and 500 ng/mL hydrocortisone, 10% FBS, and 1% penicillin/streptomycin. All cell lines were cultivated in T75 cm^3^ culture flasks (Orange Scientific, Belgium) and maintained in the incubation chamber MCO 19AIC (Sanyo, Japan), with 5% CO_2_, at 37 °C.

### 4.2. Chemicals and Cell Culture Reagents

Staurosporin (STS) (Santa Cruz, Dallas, TX, USA). Dimethyl suphoxide (DMSO), MTT, cholera toxin, insulin, and hydrocortisone (Sigma Aldrich, St. Louis, MO, USA). Resazurin (Cayman, Ann Arbor, MI, USA). DMEM without glutamine and without phenol red, Trypsin/EDTA, and FBS (Biochrom KG, Berlin, Germany). DMEM/F12 medium without phenol red (GE Healthcare, Chicago, IL, USA). All other reagents and chemicals used were analytical grade.

Stock solutions of STS and preussin (**1**) were prepared in DMSO, MTT was prepared in Phosphate buffer saline (PBS) at a final concentration of 5 mg/mL, and resazurin in PBS at 1 mM. All the stock solutions (except resazurin) were kept at –20 °C before use.

### 4.3. Preussin *(**1**)*

Preussin (**1**) was isolated from a marine derived fungus: *A. candidus* KUFA 0062, associated with the marine sponge *Epipolasis* sp. from the coral reef at the Similan Island National Park in Phang-Nga province, Southern Thailand. Isolation, purification, and characterization of preussin (**1**) have been recently reported by Buttachon et al. [21].

### 4.4. Cell Exposures

Cell suspensions were obtained by trypsinization of confluent flasks using 0.25% Trypsin/0.02% EDTA at 37 °C until cell detachment. After trypsin stopping action, cell suspensions were counted using a Neubauer chamber. Subsequently, cells were plated in different culture plates with different densities according to the type of the culture: 2D or 3D cultures. For all experiments, the final concentration of DMSO in the medium was 0.1% (v/v) and the controls received only 0.1% DMSO. All assays were performed in four independent experiments, in duplicate for each exposure condition.

#### 4.4.1. Exposure in 2D Culture

Cells were plated in 96-multiwell culture plates (Orange Scientific, Belgium) at a density of 1.0 × 10^5^ cells/mL, 100 µL/well, and kept in the incubator at 37 °C and 5% CO_2_ for 24 h for adhesion. Cells were then exposed 72 h to preussin (**1**) at different concentrations (10, 25, 50, and 100 μM), or STS (1 μM) as a positive control for apoptosis induction.

#### 4.4.2. Exposure in 3D Culture

Cells were seeded in 96-well ultra-low attachment spheroid plates (Corning, New York, NY USA, at the following densities: MCF7, MDA-MB-231 and SKBR3 40 × 10^4^ cells/mL, and MCF12A 20 × 10^4^ cells/mL, 200 µL/well. Plates were centrifuged in a centrifuge Rotina 380 R (Hettich, Germany) 200 g for 10 min and placed in the incubator at 37 °C and 5% CO_2_ for 72 h for the MCA formation. MCAs were exposed 96 h to preussin (**1**) (50 and 100 μM) or STS (1 μM).

### 4.5. Analysis of Cell Viability

#### 4.5.1. 2D Culture

##### MTT Assay

T cytotoxic effect of preussin (**1**) in breast cell lines was assessed by MTT reduction assay as described previously [82], based on reduction reaction of tetrazolium salt, pale yellow salt, forming formazan dark blue product which are dissolved and read by absorbance. This absorbance is proportional to the number of live cells, as only live cells are able to cleave MTT [83].

Briefly, after 72 h of treatment, MTT solution was added at a final concentration of 0.5 mg/mL (10 times the dilution of the stock solution) and incubated for 2 h in 5% CO_2_ at 37 °C. Exposure medium was then aspirated and the formazan crystals were dissolved by adding 150 µL of DMSO: ethanol solution (1:1) (v/v), followed by 15 min with mild agitation. Absorbance (A) was measured at 570 nm in a microplate reader Multiskan GO (Thermo Fisher Scientific, Waltman, MA, USA) [74]. Results are expressed as a percentage of cell viability relative to the solvent control (cells incubated with culture medium with 0.1% of DMSO), and calculated in accordance with the following equation:

Cell viability (%) = [(A sample at 570 nm/A control at 570 nm)] × 100

#### 4.5.2. 3D Culture

MTT and resazurin are metabolic assays based on reduction reactions. MTT has already been described in 2D culture. Resazurin is a blue compound, which acquires fluorescence when reduced into resorufin, which can be read by a fluorimeter. The fluorescence measurement correlates to the number of viable cells [84]. On the contrary, LDH (lactate dehydrogenase) assay is related to the cell membrane integrity. If the membrane is damaged, it permits the leak of LDH from the cytoplasm to the extracellular medium [48].

##### MTT Assay

MTT in 3D culture was performed according to the method previously described for 2D culture [82], with minor adaptations as MCAs must be transferred from the conical plate (where their formation and exposure occurred) to a new flat bottom 96-well plate, and with a longer incubation time. After removing the medium, formazan crystals were dissolved with DMSO (only). Absorbance measurements and the calculations were carried out in exactly the same manner as in the 2D culture.

##### Resazurin Reduction Assay

In the same way as performed for MTT, before starting the protocol, MCAs were transferred to a flat-bottom 96-well plate. Subsequently, resazurin was added to each well to a final concentration of 10 µM (100 times dilution of the stock solution). Plates were incubated for 4 h, with 5% CO_2_ and at 37 °C. Fluorescence (F) was read using excitation wavelength at 560 nm and emission wavelength at 590 nm, in a plate reader Synergy H1 (Biotek, Winooski, VT, USA) [48]. Results are expressed as the percentage of cell viability relative to the solvent control (medium culture with 0.11% DMSO), calculated in accordance with the following equation:

Cell viability (%) = [(F sample)/F control)] × 100

##### Lactate Dehydrogenase (LDH) Assay

LDH release from damaged cells in MCAs was evaluated as a biomarker for cellular cytotoxicity and cytolysis. Cell culture medium was transferred to a flat-bottom 96-well plate, and then the LDH release was detected following the manufacturer’s instructions of the Pierce™ LDH Cytotoxicity Assay Kit (Thermo Fisher Scientific, Walthman, MA, USA). The lactate produced was detected by measuring the absorbance (A) at 490 nm and 680 nm. Results are expressed as the percentage of LDH release relative to the control, calculated in accordance with the following equation:

LDH Release (%) = [(A sample at 490 nm − 680 nm)/(A control at 490 nm − 680 nm)] × 100

### 4.6. Analysis of Cell Proliferation

#### 4.6.1. 2D Culture

##### BrdU Assay

Effects on cell proliferation were evaluated by BrdU assay using the Cell Proliferation ELISA, BrdU (colorimetric) (Roche, Switzerland), according to manufacturer’s instructions as described previously [85]. Briefly, BrdU was incorporated in the place of thymidine into the DNA of cell under division. This BrdU was detected by an antibody anti-BrdU conjugated with peroxidase, the reaction product of the enzyme with the given substrate was quantified by measuring its absorbance [86,87]. The absorbance (A) was measured at 370 nm and 492 nm in the microplate reader Multiskan GO (Thermo Fisher Scientific, Walthman, MA, USA). Results are expressed as a percentage of cell proliferation relative to the control, calculated in accordance with the following equation:

Cell proliferation (%) = [(A sample at 370 nm − 492 nm)/(A control at 370 nm − 492 nm)] × 100

#### 4.6.2. 3D Culture

The BrdU assays of spheroids in 3D were performed in the same manner as that performed in 2D, with minor modification.

### 4.7. Analysis of Cell Morphology in 3D Culture

#### 4.7.1. MCA Measurements

Each MCA (one/well) was observed and photographed with an Olympus SZX10 stereomicroscope, equipped with a digital camera DP21 (Olympus, Tokyo, Japan), in two different moments: (1) Before exposure (at 72 h of formation); and (2) immediately before being sampled (after 96 h of exposure). Images were analyzed using free download AnaSP software [43]. For this, binary images were generated to measure three parameters: Area, sphericity, and solidity. For these measurements, all multicellular cell aggregates were considered, even if afterwards they were used for cell viability or other assays, accounting for a total of 12 spheroids/condition/experiment.

#### 4.7.2. Histological Analysis

MCAs were fixed in Eppendorf tubes with 10% buffered formalin (Bioptica, Milan, Itlay) for 24 h, then embedded in Histogel (Thermo Scientific, Walthman, MA, USA), according to manufacturer’s instructions, and processed for paraffin embedding in tissue cassettes using an automatic tissue processor Leica TP120 (Leica, Nussloch, Germany). The routine processing protocol consisted of the following sequence of reagents (1 h each): 70% ethanol; 90% ethanol; 96% ethanol; absolute ethanol; absolute ethanol; absolute ethanol:xylene (1:1); xylene; xylene; liquid paraffin; and liquid paraffin. Embedding was performed in an embedding station EG 1140H (Leica, Germany). Sections (3 µm) were obtained in a Leica 2255 microtome (Leica, Germany), placed onto KP-frost slides (Klinipath, Duiven, The Netherlands ), and left for 20 min in a 60 °C oven. Obtained slides were divided for standard HE staining or ICC. For HE, slides were deparaffinized for 10 min (twice) in xylene, hydrated in a decreasing series of ethanol, 5 min each step (100%, 95%, 70%), and finally running tap water, stained with Mayer’s hematoxylin (Merck, Darmstadt, Germany) for 3 min, washed with tap water for 5 min, stained with 1% aqueous eosin Y (Merck, Darmstadt, Germany), followed by quick dips in distilled water. In order to obtain definitive preparations, slides were dehydrated in an ascending series of ethanol, 5 min each (100%, 95%, 100%, 100%), cleared in xylene, and mounted using the resinous mounting medium Entellan (Merck, Darmstadt, Germany).

#### 4.7.3. Electron Microscopy

MCAs were fixed in Eppendorf tubes with 2.5% glutaraldehyde in sodium cacodylate-HCl buffer (0.15 M, pH 7.2), for 2 h at 4 °C, then washed twice with the same buffer, 10 min each. Post-fixation was performed in 1% osmium tetroxide in the same buffer as glutaraldehyde, for 2 h at 4 °C. Following the routine cell processing for electron microscopy, cells were dehydrated (30 min each step): 50%; ethanol; 70%, ethanol; 95% ethanol; absolute ethanol; absolute ethanol; propylene oxide; and propylene oxide. For epoxy resin embedding (1 h each): Successive mixture of propylene oxide and epoxy resin (respectively, 3 parts:1 part; 1 parts:1 part; 1 part:3 parts) and only resin, for the resin to penetrate gradually in MCAs. Then, embedding was performed in rubber molds, placed in a 60 °C oven for 48 h for resin polymerization. Semi-thin and ultra-thin sections were obtained in an ultramicrotome EM UC7 (Leica, Nussloch, Germany). Ultra-thin sections (≈90 nm thick) were obtained with a diamond knife (Diatome, Nidau, Switzerland), placed onto 200 mesh copper grids (Agar Scientific, Stansted, UK), and contrasted with 3% aqueous uranyl acetate (20 min) and Reynold’s [88] lead citrate (10 min). Grids were observed in the transmission electron microscope JEOL 100CXII (JEOL, Tokyo, Japan), operated at 60 kV, and photographed with the Orius SC1000 CCD digital camera (Gatan, Pleasanton, CA, USA).

### 4.8. Immunocytochemistry in 3D Culture

Sections were deparaffinized and hydrated following the sequence of HE staining. The next step consisted of heat antigen retrieval that was performed in a pressure cooker, using citrate buffer (0.01 M, pH 6.0) for 2 min after reaching the maximum pressure. Later, the slides slowly cooled, and endogenous peroxidases were blocked with 3% hydrogen peroxide in methanol (10 min). The excess of hydrogen peroxide was removed by washing in tris-buffered saline (TBS), pH 7.6 (5 min).

In order to save reagents, water was removed around the sections (without letting them dry) so that a hydrophobic pen could be applied (Leica, Germany). In the sequence, unspecific reactions were blocked using the blocking reagent from the Novolink™ Polymer Detection System kit (Leica Biosystems, Nussloch, Germany) (5 min), followed by two washes in TBST (5 min); that is, in the above-mentioned TBS we added 0.05% of Tween 20 (Sigma, St. Louis, MO, USA). The incubation with primary antibodies was overnight (16 h), using a humidified chamber at 4 °C. Primary antibodies applied were diluted in PBS with 5% bovine serum albumin (BSA) (Nzytech, Lisbon, Portugal). We applied two primary antibodies: Rabbit monoclonal anti-Ki67, clone SP6 (Biocare Medical, Pacheco, CA, USA), dilution of 1:200, for assessing cell proliferation; and rabbit polyclonal anti-caspase-3, ab 13847 (Abcam, Cambridge, United Kingdom), diluted 1:5000, for assessing caspase dependent apoptosis.

The signal amplification and revelation were performed with the Novolink™ Polymer Detection System (Leica Biosystems, Nussloch, Germany) according to the manufacturer’s instructions. Slides were counterstained with Mayer’s hematoxylin for one min, washed in tap water, dehydrated, and mounted. Observations using an Olympus BX50 light microscope (Olympus, Tokyo, Japan). and photographs were obtained with a digital camera DP21 (Olympus, Tokyo, Japan).

### 4.9. Statistical Analysis

Descriptive and inferential statistics were performed using GraphPad Prism 6.0 software (GraphPad Software, La Jolla, CA, USA). The results are expressed as mean ± standard deviation (SD) of four independent experiments. Significant differences (*p* ≤ 0.05) were assessed by one-way ANOVAs, followed by the post-hoc Holm- Šídák multiple comparison test whenever the ANOVA disclosed significant results for the tested effects. The normality and homogeneity of variance were confirmed by the Shapiro-Wilk test and the Levine test, respectively.

## 5. Conclusions

The results obtained in this study are very interesting, since preussin (**1**)-induced cytotoxic and antiproliferative effects on a panel of human breast cancer cell lines when cultured in 2D and 3D were observed. The effects varied according to the cell line molecular characteristics. The results obtained in the 3D model followed the same tendency as those found in the 2D model; however, cells in the 3D model showed more resistance to the impact of preussin (**1**). In this regard, the use of a multi-endpoint approach, which included histological evaluations, was important. The cytotoxic activity of preussin (**1**) in non-tumor cells was also an important point, since, in future studies, strategies to decrease the side effects of preussin (**1**) in non-target cells should be evaluated. Overall, the data support the potential of preussin (**1**) as a scaffold for the development of an anticancer drug candidate and call for further fundamental studies in vitro to clarify the molecular targets and the signaling pathways involved in the anticancer activity demonstrated by preussin (**1**).

## Supplementary Materials

The following are available online at https://www.mdpi.com/1660-3397/17/8/448/s1, Figure S1: Effect of preussin (**1**) (50 and 100 µM) in the MCA sphericity (left side) and solidity (right side) 3D culture. (A, B) MCF7, (C, D) MDA-MB-231, (E, F) SKBR3 and (G, H) MCF12A cells after 96 h of incubation. Cells treated with 0.1% DMSO (SC) and STS at 1 µM were included as negative and positive control, respectively. The results were expressed as the percentage of cell viability relative to negative control and are presented as mean ± standard deviation (SD) of four independent experiments (twelve duplicates per replica).
