# Supplementary material for: Cytotoxic and Antiproliferative Effects of Preussin, a Hydroxypyrrolidine Derivative from the Marine Sponge-Associated Fungus Aspergillus candidus KUFA 0062, in a Panel of Breast Cancer Cell Lines and Using 2D and 3D Cultures
Source: Mar Drugs. 2019 Jul 30;17(8):448. doi: 10.3390/md17080448 (PMC6722565; doi:10.3390/md17080448)
Supplement: Supplementary file 1 [file marinedrugs-17-00448-s001.zip › marinedrugs-563368-supplementary.pdf]

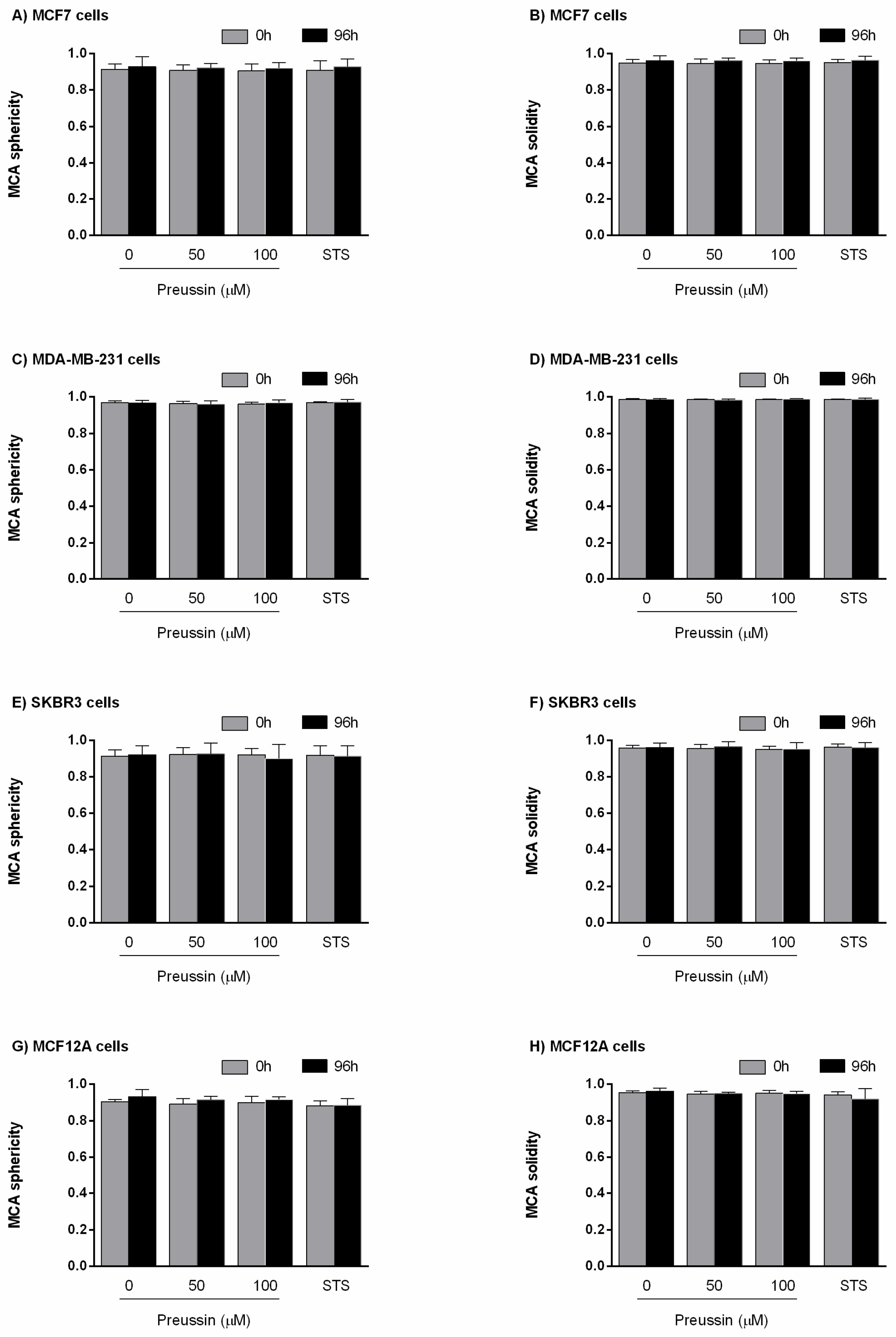

Figure S1. Effect of preussin (50 and 100 μM) in the MCA sphericity (left side) and solidity (right side) 3D culture. (A, B) MCF7, (C, D) MDA-MB-231, (E, F) SKBR3 and (G, H) MCF12A cells after 96 h of incubation, Cells treated with 0.1% DMSO (SC) and STS at 1 μM were included as negative and positive control, respectively. The results were expressed as the percentage of cell viability relative to negative control and are presented as mean ± standard deviation (SD) of four independent experiments (twelve duplicates per replica).
